# Efficient iterative CRISPR/Cas9 editing using *sid-1* co-conversion and feeding RNAi in *Caenorhabditis elegans*

**DOI:** 10.1093/g3journal/jkaf128

**Published:** 2025-06-06

**Authors:** Alexandra S Weisman, Nicole M Fisher, Craig P Hunter

**Affiliations:** Department of Molecular and Cellular Biology, Harvard University, 16 Divinity Avenue, Cambridge, MA 02138, USA; Department of Molecular and Cellular Biology, Harvard University, 16 Divinity Avenue, Cambridge, MA 02138, USA; Department of Molecular and Cellular Biology, Harvard University, 16 Divinity Avenue, Cambridge, MA 02138, USA

**Keywords:** *C. elegans;* CRISPR/Cas9, co-conversion, *sid-1;* iterable, sequential, cyclical, serial, revertible, interconvertible, WormBase

## Abstract

We present a *sid-1* loss-of-function and restoration-of-function CRISPR/Cas9 co-conversion protocol in *Caenorhabditis elegans*. Introducing CRISPR reagents that induce *sid-1* loss-of-function can produce survivors on lethal RNAi foods while reagents that induce *sid-1* restoration-of-function can be screened for restoration of visible RNAi phenotypes. Both methods efficiently reduce the pool of candidates from hundreds or thousands of F1 progeny to tens with minimal experimenter effort. Furthermore, our optimized *sid-1* CRISPR design allows a high ratio of CRISPR reagents targeting the gene of interest, maximizing successful co-conversion events. The interconvertibility of the *sid-1* locus readily enables this strategy to be leveraged to iteratively create complex strains with multiple gene edits.

## Introduction

Co-CRISPR and co-conversion are widely used in *Caenorhabditis elegans* genome editing to confirm reagent functionality and identify animals likely to contain successful edits of interest. However, many of these strategies introduce mutations whose phenotypes complicate downstream analyses and thus must be removed prior to subsequent experiments (Arribere *et al*. 2014; Kim *et al*. 2014). Rescue of temperature-sensitive *pha-1(e2123)* restores the wild-type sequence but is only compatible with strains that can tolerate 25°C growth (Ward 2015). A recent publication employs an iterative, or “flip-flop”, Dumpy/non-Dumpy selection procedure, yet one must then inject Dumpy animals (Lins *et al*. 2023). These challenges can make co-CRISPR/conversion schemes inefficient for iterative or complex genome-editing workflows.

We have developed highly efficient *sid-1* Cas9 cleavage and repair reagents that make *sid-1*, when combined with feeding RNAi, a highly effective co-conversion marker. Feeding RNAi is a widely used technique, and the food-dependent phenotype can be tailored to experimenter needs and experience. Loss-of-function (LOF) mutations in *sid-1* confer RNAi resistance, providing a clear, selectable phenotype, while restoration-of-function (ROF) *sid-1* alleles restore RNAi sensitivity, providing numerous choices for visible phenotypes (Winston *et al*. 2002). These phenotypes can be assessed rapidly in the F1 and F2 progeny, providing immediate feedback on the preparation and injection of the CRISPR reagents. If *sid-1* co-conversion succeeds but a co-injected edit of interest (EOI) fails, the issue is likely due to guide RNA or repair template design rather than reagent handling. Additionally, identifying candidates with precise edits among hundreds of offspring is time-consuming and labor-intensive. Our findings demonstrate that the vast majority of animals with *sid-1* co-conversions also harbor the desired edit in the gene of interest, significantly reducing the number of progeny that must be screened. Finally, the *sid-1* locus can be readily and rapidly restored to wild-type function, eliminating the co-CRISPR phenotype and/or enabling the introduction of additional EOIs. This reiterative cycling between *sid-1* LOF and s*id-1* ROF enables generating complex genome edits. By combining simplicity, reliability, and versatility, *sid-1* co-conversion offers a transformative approach for genome editing in *C. elegans*, facilitating both single and sequential edits with minimal technical barriers.

## Materials and methods

### *C. elegans* maintenance

Strains were maintained at 20°C on normal growth medium plates seeded with OP50 (Brenner 1974).

### Design and optimization considerations of CRISPR guides and repair templates

One (or 2) CRISPR RNA (crRNA) and a single-stranded oligodeoxynucleotide (ssODN) repair template containing two ∼35 nucleotide (nt) homology arms were used for LOF and ROF cassettes. When possible, target sequences with the recommended NG nucleotides upstream of the protospacer adjacent motif (PAM) were selected for higher editing efficiency (Farboud *et al*. 2019). See Supplementary file for exact sequences of crRNAs and ssODN repair templates.

*sid-1* LOF alleles (*qt160* and *qt164*, see Supplementary file) were generated by a crRNA (*sid-1* AG) that targets early in the wild-type sequence and a 117 nt anti-sense ssODN repair template. Design elements from the Meyer, Seydoux, and Sternberg groups were combined to maximize the theoretical efficiency of the *sid-1* LOF repair (Paix *et al*. 2016, 2017; Wang *et al*. 2018; Farboud *et al*. 2019). Both *sid-1* LOF homology arms are proximal/terminal, immediately abutting the double-stranded DNA break (Paix *et al*. 2016). As in Wang *et al*. (2018), the repair template directs insertion of a universal cassette containing an exogenous Cas9 target sequence, stop codons in all 3 frames, and a restriction site, as well as inducing a downstream frameshift. Our modifications include the following: (1) switching the *NheI* site to *KpnI* to permit enzyme activity directly in Phusion PCR buffer, (2) further optimizing the inserted exogenous crRNA target sequence from Paix *et al*. (2017) (UcrRNA_AW1) to increase the off-target score without affecting the on-target score (Integrated DNA Technologies 2019, RRID:SCR_025813), (3) changing the 2 nucleotides upstream of the PAM from AG to GG to improve ribonucleoprotein (RNP) cutting efficiency, and (4) inverting the orientation of the exogenous crRNA target sequence within the cassette to improve the repair efficiency of subsequent *sid-1* ROF edits: inversion relative to the Wang *et al*. (2018) STOP-IN cassette consolidates the recessed homology to one arm (6 nt and 39 nt recession instead of 17 nt and 28 nt).

To convert *sid-1* LOF to *sid-1* ROF (wild-type), a crRNA targeting the inserted exogenous target sequence (UcrRNA_AW1) and a 73 nt ssODN consisting entirely of homology arms were used.

The *mex-6* deletion used 2 crRNAs targeted near the start codon (*mex-6* ATG) and within the 3′ UTR (*mex-6* UTR) (Sternberg *et al*. 2024). A 107 nt ssODN repair template directed insertion of a stop cassette and our exogenous crRNA (UcrRNA_AW1) target sequence to produce a precise 2219 nt deletion.

*him-8* was targeted for insertion of a reversible *LOF* cassette, similar to the *sid-1* LOF cassette, by injecting a single crRNA (*him-8* AA) and a 108 nt ssODN repair template (Sternberg *et al*. 2024). To convert *him-8* LOF to *him-8* ROF (wild-type), a crRNA targeting the inserted exogenous target sequence (UcrRNA_AW1) and a 67 nt ssODN consisting entirely of homology arms were used.

### CRISPR reagent preparation

We generally followed the Star Protocol from the Mello group and recommend using that as a detailed reference (Ghanta *et al*. 2021). Our modifications to their protocol include: (1) ssODN repair template concentration (1 µL of 40 pmol/μL stock was added), (2) following assembly of the full injection mix, we centrifuge at 13,000*×g* for 2 minutes to avoid needle clogging, transfer 19 μL to a fresh tube and keep on ice, (3) 0.5 μL of mixture was loaded into an injection needle and injections were performed until at least 3 high-quality injections (of both gonad arms) have been achieved. After centrifugation, *sid-1*:EOI reagents were combined at a 1:9 ratio. Reagent mixtures were stored at 4°C. Animals were recovered on a common plate for 6 hours before transferring individual P0 s to fresh plates.

### Stringent needle hygiene

CRISPR RNP injection needle hygiene begins with stringent chain of custody of capillaries: new capillaries (freshly opened from manufacturer) were aliquoted in small batches into 15 mL polypropylene screw cap tubes while wearing gloves that were treated with RNase-away (or similar product). A similarly treated brand-new razor blade was used to cut a hole in the cap just large enough for a single capillary to slide through. Aliquoted capillaries were stored in an RNase-free area.

Prior to pulling injection needles, a Kimwipe wetted with RNase-away was used to swab all portions of the needle puller that may contact the capillary. After 1 minute of contact time, the same portions of the puller were rinsed with a Kimwipe wetted with RNase-free water, then dried. We similarly treated the needle holder on the injection rig. Since our puller and rig are also used for non-RNase-free injections, we repeated this procedure each time prior to pulling new needles.

Care was taken to only handle capillaries and pulled needles with RNase-away treated gloves, and only in the middle; we did not touch the ends. A fresh capillary was shimmied through the hole in the cap of the tube, loaded into the needle puller, and the program was run. The freshly pulled needles were rested on clay supports in an RNase-away treated petri dish and humidified by a Kimwipe saturated with RNase-free water. Good RNase-free technique and tips were used to load needles with injection mix (we backloaded quick-fill filamented capillaries, 1B100F-4, World Precision Instruments (RRID:SCR_008593)). Loaded needles were fitted into the needle holder of the injection rig with RNase-away treated gloves. From this point on standard (casual) injection techniques were used (Halocarbon/paraffin oil, buffers), after all, worms are not RNase-free.

### Injection considerations to facilitate troubleshooting

When possible, we followed F1 offspring from P0 bearing F1 roller frequencies above 5% and moderate fertility (∼80–200 offspring over 48 hours) but note that we also recovered co-CRISPR and edits of interest from animals with no F1 rollers or low fertility (as few as 18 offspring). Since the *sid-1* ROF approach facilitates screening of the whole F1 brood it obviates identification of the cohort within the brood that was exposed to the injection mix (rollers). However, users may still wish to include the *pRF4::rol-6* plasmid to distinguish issues with microinjection technique from issues with reagent preparation (Mello *et al*. 1991).

### RNAi food and plate preparation, storage and use

RNAi foods with post-embryonic RNAi phenotypes were selected to avoid confounding results from maternal effects. Clones for *act-5/*Y25C8.2, *unc-22/*ZK617.1 were sourced from the Ahringer Library (Kamath and Ahringer 2003; RRID:SCR_017064). RNAi food was prepared as in Protocol 1 (Kamath *et al*. 2001) except with 100 µg/mL carbenicillin (Carb) and given 24-hour drying and induction time on isopropyl-β-D-thiogalactopyranoside (IPTG)/Carb plates. Note that IPTG/Carb plates have a limited 4°C shelf life and should be seeded within 1 month after being poured and should be used within 8 weeks of pouring. Whenever RNAi plates are prepared, immediately test wild-type and *sid-1* mutant controls to confirm the RNAi food is properly prepared before proceeding with phenotypic scoring within *sid-1* co-conversion workflows. Assays were performed at 20°C.

### *sid-1* LOF workflow using *act-5* RNAi resistance

After injected P0 animals have laid progeny on 2 OP50 plates (the first day post-injection and the second day post-injection), score the plates for the proportion of roller progeny. Select 2–4 P0s with the highest roller proportions and moderate fertility. Move the F1 progeny to *act-5* RNAi plates when they are L4s to avoid *act-5* effects on fertility. It is advisable to single as many F1s as possible to increase the likelihood of recovering edits of interest. Some experiments in this study utilized pools of 2 to 5 F1s at this step to increase the number of F1s scored while maintaining a practical number of RNAi plates (pooled experiments are indicated in figure legends and Supplementary file), in these cases, only a single F1 line was followed from each plate. After 24 hours, remove the adult to produce a semi-synchronized cohort for facile scoring 2 to 3 days later. Identify *act-5* RNAi-resistant animals by comparison to age-matched positive (homozygous *sid-1* mutant) and negative (wild-type) controls. Only homozygous *sid-1* mutants will grow past the L3 stage; therefore, plates with multiple L4/young adults are likely successful co-conversion lines. Single 2–4 sibling F2s from each *act-5* resistant plate to fresh plates; once these F2 animals have laid sufficient progeny, the F2 adults can be used directly for PCR genotyping to identify homozygous *sid-1* and EOI.

We note that if only lines with imprecise *sid-1* edits and precise EOI edits are found, imprecise *sid-1* edits can be easily outcrossed using non-lethal RNAi food. If additional *sid-1* co-conversion is being used, first confirm *sid-1* LOF as a precise insertion with an intact exogenous crRNA target site and PAM by sequencing to ensure it can be converted to ROF.

### *sid-1* ROF workflow using *unc-22* RNAi sensitivity

Recovered (6 hours) injected P0s were singled onto *unc-*22 RNAi plates and transferred on day 2 to a second *unc-*22 RNAi plate for another day and removed. Candidate RNAi-sensitive (twitching) L4 to young adult animals were identified by comparison to positive (wild-type) and negative (homozygous *sid-1* mutant) controls. F1 twitchers were singled to produce a subsequent generation on *unc-22* RNAi plates. It is advisable to single as many F1s as possible to increase the likelihood of recovering edits of interest. In our hands, well-made *unc-22* RNAi plates do not require addition of levamisole to identify twitchers, but animals can be picked into 3 mM levamisole to facilitate scoring (Lewis *et al*. 1980). To identify potential homozygous *sid-1* and EOI, and avoid loss of non-phenotypic EOI edits, isolate ∼12 twitching sibling F2s from each *unc-22* F1 plate. Once F2 animals have laid sufficient progeny, the adult F2 can be used directly for PCR genotyping. Once homozygous ROF animals (100% twitching progeny) are identified, RNAi food is no longer required.

### Him phenotype scoring

L4 animals were singled and allowed to lay for 24–48 hours and then removed. Scoring the sex of 25–35 adult self-progeny was sufficient to call animals Him or Non-Him. Twelve to 16 F2s per candidate *him-8* LOF F1 line were singled to increase the likelihood of recovering homozygous animals that exhibit the Him phenotype.

### Band shift analysis and sequencing

F2 animals were used for band shift analysis and F2 or F3 apparent homozygotes were used for Sanger sequencing. Individual adults were lysed as in Minkina and Hunter (2017), except 15 µL of buffer was used per adult and the 95°C step was extended to 15 minutes. PCR was performed in Phusion HF Buffer (New England Biolabs; RRID:SCR_013517) scaled to 20 µL reactions, 3 µL of the worm lysate as template and a standard 30-cycle 3-step PCR (see Supplementary file for cycling parameters, oligo sequences, and amplicon lengths). The precise edit produced by our *sid-1* LOF reagents generates a 44 base pair (bp) band shift compared to wild-type and thus is easily visualized by gel electrophoresis on a 2% Agarose/TBE gel. Upon efficient *KpnI* digestion, wild-type *sid-1* amplicons are unchanged, but *sid-1* LOF amplicons are cleaved once. This optional digestion step can provide further insight on the precision of repair and inform selection of candidates for sequencing. The candidates selected for sequencing in this study were analyzed without digestion.

The precise *mex-6* deletion produces a 2219 bp band shift compared to wild-type. The precise edit our *him-8* LOF reagents produce has a 41 bp band shift compared to wild-type.

### Sequencing off-target genes

Animals were lysed as above. Four F2s were collected per candidate F1. Quality scores and continuous read length were used to identify poor quality runs; furthermore, individual base quality scores were examined within a 100 nt window of the putative cut site using a custom R script (see data availability statement): runs with any individual quality score <40 within that window were re-run and the traces were manually reviewed. Results from the highest quality run for each F2 are available in the Supplementary file.

### Guidance on naming any alleles generated with our co-CRISPR reagents

If using strain HC1292 to convert *sid-1(qt160)* LOF to ROF, which is molecularly wild-type, the resulting strain should be named sid-1(YourLabAlleleDesignation##[*qt160]) (Tuli *et al*. 2018).

Strains, plasmids, crRNAs, ssODN repair templates, and oligos used in this study are listed in the Supplementary file.

## Results

### *sid-1* loss-of-function and restoration-of-function strategy

**Fig. 1.**
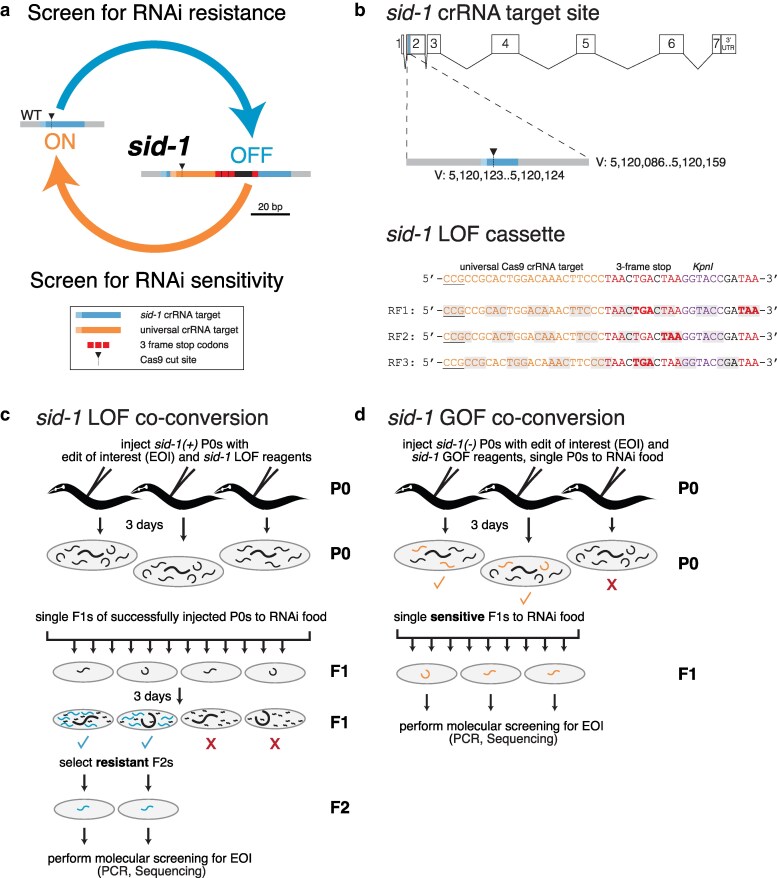
The iterable *sid-1* co-conversion strategy provides a flexible system that enables sequential editing. a) Overview of iterable co-conversion strategy. b) Genomic location of the endogenous *sid-1* crRNA target site and sequence of the loss-of-function cassette; all 3 reading frames (RF) are presented. Workflow of c) *sid-1* loss-of-function (LOF) and d) restoration-of-function (ROF) co-conversions.

We modified design elements from previously described methods (Paix *et al*. 2016, 2017; Wang *et al*. 2018; Farboud *et al*. 2019) to create a highly efficient and reversible *sid-1* allele (Fig. 1a). See methods section for full details and advice on EOI design. The *sid-1* LOF was generated by a crRNA that targets the wild-type sequence and a 117 nt ssODN that templates insertion of an exogenous crRNA target site and 3-frame stop knock-in cassette (Fig. 1b and Supplementary file). To convert *sid-1* LOF to *sid-1* ROF (wild-type), a crRNA that targets the exogenous sequence and a 73 nt ssODN repair template were used. Once the wild-type sequence has been restored, further co-conversion edits are enabled (Fig. 1a). Our screening pipelines begin with either the LOF or ROF RNAi phenotype, proceeds to a PCR band shift assay to identify programmed insertion/deletions, and concludes with selective sequence confirmation.

### *sid-1* LOF CRISPR is effective and easy to identify

To identify *sid-1* LOF animals, the F1 offspring of injected N2s were moved to a post-embryonic lethal RNAi food (*act-5*) at the L4 stage (Fig. 1c). Only homozygous *sid-1* LOF animals will grow to adulthood on *act-5* RNAi plates. A single F2 adult was selected from each such plate to establish candidate F1 lines for molecular confirmation. We used PCR to identify F1 lines with the expected *sid-1* band shift and then sequenced select candidates to confirm precise insertion of the *sid-1* LOF cassette.

**Fig. 2.**
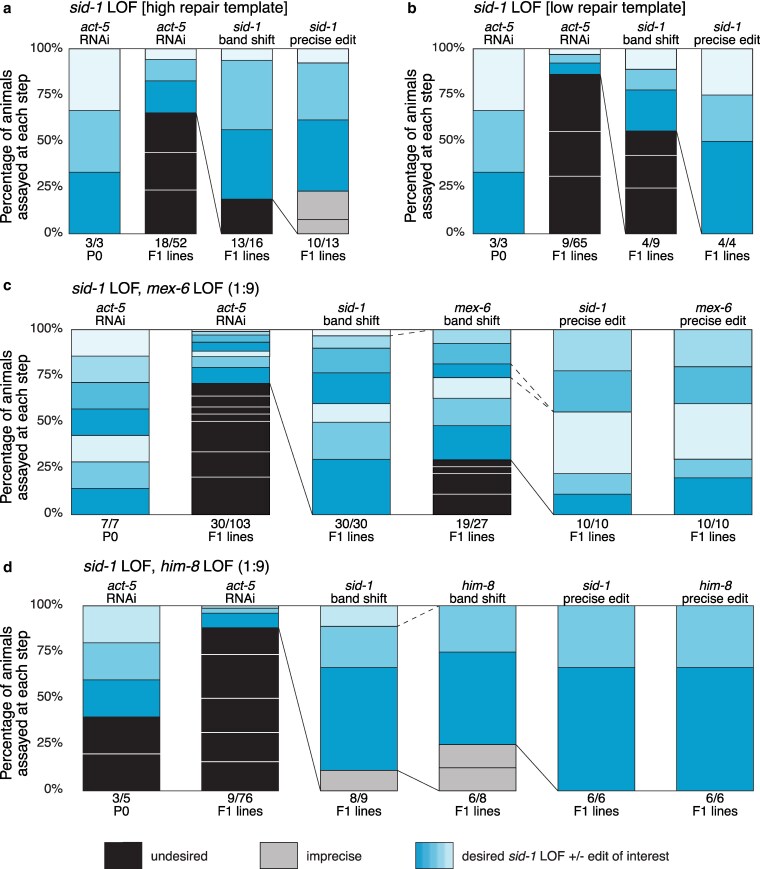
*sid-1* LOF strategy readily identifies co-conversion candidates. a–d) Each bar shows the percentage of animals of interest in blue (phenotypic or precisely edited), imprecisely edited in gray, and undesired in black (non-phenotypic or unedited animals). Each box within a bar marks an individual P0 and its progeny of interest across the screening steps. Candidates carried forward are indicated by connecting lines; dotted lines indicate a P0 cohort excluded from further analysis. F1s were singled unless otherwise indicated. Two representative experiments are shown for injection mixes containing only *sid-1* LOF reagents at high [4 pmol/µL] a) and low [0.4 pmol/µL] b) ultramer repair template concentration. Two representative *sid-1* LOF co-conversion experiments identifying candidates for *mex-6* (c) or *him-8* (d) LOF edits. The results from 2 replicate *mex-6* experiments are presented with P0s grouped by experiment: the top 4 P0s (F1s singled) and bottom 3 P0s (F1s plated in pools of 3).

We observed that 77% (*n* = 13) of candidate F1 lines had precise edits at the *sid-1* locus, not insertions or deletions (Fig. 2a). If RNAi resistance occurs but precise insertions are not observed, it likely indicates issues with the repair template. Lowering the repair template concentration to simulate co-conversion conditions reduced the number candidates but did not adversely affect the recovery of precisely edited *sid-1* alleles (Fig. 2b). All P0 animals produced at least one precise *sid-1* edit, making identification of candidate lines easy and quick. One sequence-confirmed *sid-1* LOF precise edit was designated as *qt160* and curated as strain HC1185; its outcrossed descendant, HC1292, is available at the Caenorhabditis Genetics Center (CGC, RRID:SCR_007341). Our *sid-1* crRNA has 2 potential off-target sites in the genes *linc-165* and *pxd-1*, both of which have 4 mismatches within the guide sequence (CRISPOR (RRID:SCR_015935); see Supplementary file). While it is unlikely that sites with this many mismatches would be cut, we sequenced them to be sure (Au *et al*. 2019; Farboud *et al*. 2019). Concomitant with our guide RNA design principles, we failed to detect any sequence changes within 100 nt of the putative cut sites of the off-target genes for 8 independent F1s in addition to strain HC1185 (see Supplementary file). These results suggest that when screening for edits of interest free of off-target lesions, 2 or 3 independent candidates should suffice unless one is exceedingly unlucky.

### Validating co-CRISPR of *sid-1* LOF and edits of interest

We designed CRISPR reagents to precisely edit *mex-6* and *him-8*, 2 germline-expressed genes whose loss-of-function phenotypes should not interfere with identifying or recovering edits of interest. Both genes were targeted for insertion of LOF cassettes containing a triple stop cassette, exogenous crRNA target site and *KpnI* restriction site similar to the *sid-1* LOF cassette. To increase the likelihood that *sid-1* edited animals would also harbor the edit of interest, we employed a reagent ratio of *sid-1*:EOI of 1:9, a more aggressive ratio then utilized in most other methods (Paix *et al*. 2015, 2016; Farboud *et al*. 2019; Yang *et al*. 2020).

The *mex-6* gene is functionally redundant with *mex-5*, thus it can be deleted without causing a phenotype (Schubert *et al*. 2000). *mex-6* coding sequence was replaced with the LOF cassette using 2 crRNAs and a 107 nt ssODN repair template. Precise *mex-6* EOIs were readily recovered using *sid-1* LOF co-conversion (Fig. 2c). To assess reproducibility, this experiment was performed twice, with RNP mixtures prepared several months apart; combined data are shown. In both experiments, all injected N2 animals produced F1s that laid *act-5* RNAi-resistant F2 progeny. All 30 F1 lines produced *sid-1* PCR band shifts indicating successful insertion of the repair template and the absence of in/dels at the *sid-1* locus. Nineteen F1 lines also exhibited expected *mex-6* band shifts, demonstrating deletion of the entire coding region and efficient repair template insertion at the *mex-6* locus. Ten F1 lines selected from 5 different P0s were confirmed to have precise edits at both *sid-1* and *mex-6*. In summary, 7 injected P0 animals produced 30 F1 resistant lines, 19 of which contained the expected *mex-6* band shift, and all 10 sequenced F1 lines proved to have precise edits at both *sid-1* and *mex-6*.

The *him-8* gene is required for proper X-chromosome segregation; null alleles increase the frequency of males (Hodgkin *et al*. 1979). The *him-8* coding sequence was disrupted by inserting an LOF cassette using a single crRNA and a 108 nt ssODN to direct repair. Precise *him-8* EOIs were readily recovered using *sid-1* LOF co-conversion (Fig. 2d). Three of 5 injected N2 animals produced 9 F1-resistant lines, 8 of which showed the expected *sid-1* band shift. Six of these 8 exhibited the expected *him-8* band shifts, and all 6 F1 lines had precise edits at both *sid-1* and *him-8*. One sequence-confirmed isolate was curated as HC1189 (*him-8(qt163) IV; sid-1(qt164) V*). All lines with precise *him-8* LOF edits displayed the Him phenotype.

Our results demonstrate that precise *sid-1* edits and EOI occur more frequently than imprecise edits and frequently occur together.

### Stringent needle hygiene increases editing efficiency

**Fig. 3.**
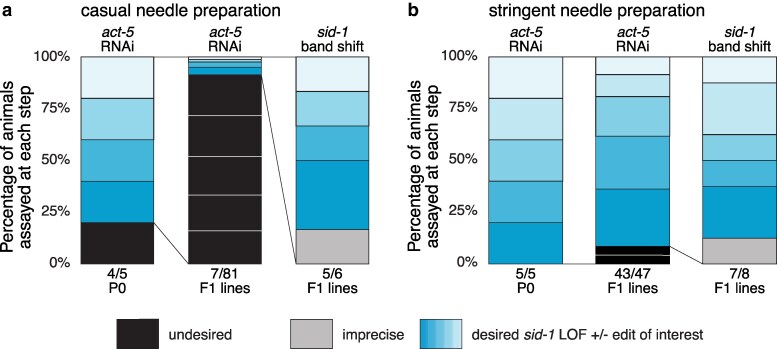
Needle preparation affects editing efficiency but not proficiency. a, b) Each bar shows the percentage of animals of interest in blue (phenotypic or precisely edited), imprecisely edited in gray, and undesired in black (non-phenotypic or unedited animals). Each box within a bar marks an individual P0 and its progeny of interest across the screening steps. Candidates carried forward are indicated by connecting lines; dotted lines indicate a P0 cohort excluded from further analysis. The relative efficiency of *sid-1* LOF conversion attempted with casual needle preparation (a) and stringent needle preparation (b) are shown. In both conditions the repair template concentration was 4 pmol/µL and F1s were plated in pools of 2–5.

During reagent testing, we found that needle hygiene was a critical factor that influenced CRISPR efficiency. See methods section for a description of proper needle hygiene. When less stringent needle hygiene procedures were followed, the proportion of *act-5* resistant F1 lines was dramatically reduced (Fig. 3a). Stringent needle hygiene, including RNAse-free needle aliquoting, storage, pulling, and loading, substantially increased the proportion of candidate F1 lines (Fig. 3b). Although fewer F1 candidates were identified when using casual needle conditions, editing proficiency (precise band shifts) remained high. Therefore, *sid-1* LOF co-conversion enables identification of candidate lines even when reagent preparation is suboptimal.

### *sid-1* ROF CRISPR is effective and easy to identify

**Fig. 4.**
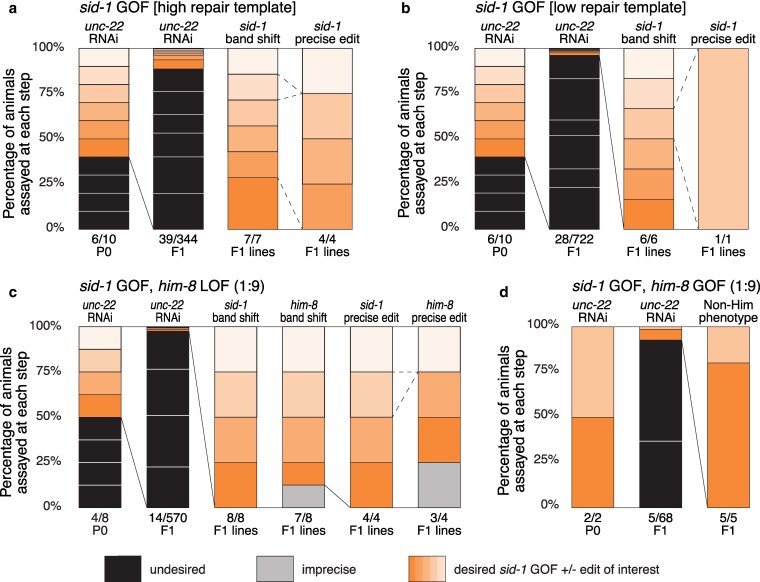
*sid-1* ROF strategy simplifies identification of co-conversion candidates. a–d) Each bar shows the percentage of animals of interest in orange (phenotypic or precisely edited), imprecisely edited in gray, and undesired in black (non-phenotypic or unedited animals). Each box within a bar marks an individual P0 and its progeny of interest across the screening steps. Candidates carried forward are indicated by connecting lines; dotted lines indicate a P0 cohort excluded from further analysis. Two representative experiments are shown for injection mixes containing only *sid-1* ROF reagents at high [4 pmol/µL] a) and low [0.4 pmol/µL] b) ultramer repair template concentration. c) *sid-1*(*qt160*) animals were injected with reagents to insert a revertible *him-8* LOF cassette. d) *him-8(qt163); sid-1(qt164)* animals were injected with reagents to revert both loci to wild-type; only phenotypes were scored.

Since a single wild-type copy of *sid-1* restores feeding RNAi sensitivity, after a brief recovery, injected animals were singled to a non-lethal RNAi food (*unc-22*) and F1 progeny were scored for twitching (see Methods) (Fig. 1d). As observed for *sid-1* LOF CRISPR, repair template concentration correlates with the number of recovered candidates but does not affect the ability to recover precise *sid-1* ROF alleles (Fig. 4a and 4b). Our *sid-1* ROF *unc-22* results highlight the power of this co-conversion paradigm; simply by scoring for visible twitching, the number of probable candidates were reduced 10- to 20-fold. Our exogenous crRNA has 2 potential off-target sites in the genes *clec-83* and *faxc-1*, both of which have 4 mismatches within the guide sequence (CRISPOR (RRID:SCR_015935); see Supplementary file). While it is unlikely that sites with this many mismatches would be cut, we sequenced them to be sure (Au *et al*. 2019; Farboud *et al*. 2019). We failed to detect any sequence changes within 100 nt of the putative cut sites of the off-target genes for 7 independent F1s, thus 2 or 3 independent candidates should suffice unless one is exceedingly unlucky (see Supplementary file).

### Validating co-conversion of *sid-1* ROF and edits of interest

To validate *sid-1* ROF co-conversion, we used strain HC1185 (*sid-1(qt160)V)* and the *him-8* LOF reagents established earlier (Fig. 2d). We then used *sid-1* ROF to repair *him-8*, using strain HC1189 (*him-8(qt163) IV; sid-1(qt164) V*) and reagents that revert the *him-8* LOF allele (*qt163*) to wild-type (*him-8* ROF).

Precise *him-8* LOF alleles were readily identified and recovered among *sid-1* ROF animals (Fig. 4c). Specifically, 4 of 8 injected HC1185 (*sid-1* LOF) P0s produced 14 *unc-22*-sensitive F1s and 12 of these gave rise to *unc-22*-sensitive F2s. Eight of the confirmed 12 F1 lines produced the expected *sid-1* band shifts. Seven of these 8 F1 lines also exhibited the expected *him-8* band shifts. Four sequenced F1 lines, selected from 4 different P0s, had precise *sid-1* reversion and 3 had precise *him-8* LOF insertions. As expected, all lines with precise homozygous *him-8* LOF edits displayed the Him phenotype.

To test co-conversion, we injected HC1189 (*him-8* precise LOF, *sid-1* precise LOF) with our exogenous crRNA that targets both genes and 2 gene specific ssODNs to restore both *him-8* and *sid-1* to their respective wild-type functions. Two injected HC1189 P0s produced 5 *unc-22* sensitive F1s, all of which gave rise to *unc-22*-sensitive F2s that produced a non-Him phenotype, indicating that *him-8* function was restored and the locus was precisely reverted to wild-type (Fig. 4d).

## Discussion

The *sid-1* co-CRISPR protocol described here offers a streamlined approach for genomic editing in *C. elegans*. Feeding RNAi is inexpensive, ubiquitous, and easy. RNAi phenotypes are conditional on the provided food, thus altering RNAi sensitivity offers multiple options for screening and selection phenotypes; experimenters can select an RNAi phenotype they are experienced scoring that is compatible with their desired edits of interest. Furthermore, the high efficiency of recovering precise edits at the *sid-1* locus, likely enabled by reagent optimization including the aggressive *sid-1*:EOI concentration ratio of 1:9, is tightly coupled to the recovery of precise edits at the target locus, reducing the number of animals that must be injected, screened, and sequenced. If the *sid-1* LOF strategy is used, and *sid-1* does not affect phenotypic analysis, the edited strain can be analyzed directly, or *sid-1* can be readily reverse-edited to wild-type. Lethal RNAi, such as *act-5*, further simplifies LOF screening by removing all animals without *sid-1* editing. As LOF candidates are tested as F2s, there is no likelihood of mosaicism.

The *sid-1* ROF strategy restores the *sid-1* wild-type sequence in the F1 progeny, thus RNAi sensitive animals can be identified in the F1 generation. As ROF selects for restoration of function this strongly favors the recovery of precise edits compared to any LOF co-conversion scheme. Furthermore, the dominant phenotype identifies progeny exposed to both functional Cas9 reagents and to active *C. elegans* homology directed repair processes, which likely contributes to the efficient recovery of target gene EOIs. For ROF co-conversion, non-mosaic animals can be easily identified by testing one subsequent generation for RNAi sensitivity. For all these reasons, we strongly recommend using the strain HC1292 and the efficient *sid-1* ROF strategy for single EOI designs. To reduce the number of spontaneous mutations (from normal culturing in the lab) prior to deposition at the CGC, the *qt160* allele was outcrossed 6 times to N2. Regardless, prudent adopters may wish to sequence any loci they intend to edit and confirm wild-type behavior in any non-RNAi assays before injecting reagents.

Finally, the ease of iteratively cycling between *sid-1* LOF and ROF while creating strains with multiple alleles and low incidence of off-target editing eliminates the need to use multiple co-conversion markers or to remove co-conversion marker(s) by outcrossing. While other schemes have 1 or 2 of these benefits, our scheme simultaneously maximizes recovery of desired edits of interest, minimizes experimenter effort and maintains a degree of flexibility not available in previously described strategies. We encourage researchers interested in adopting this approach to obtain strains from the Caenorhabditis Genetics Center (RRID:SCR_007341) and reach out for additional guidance.

## Supplementary Material

jkaf128_Supplementary_Data

## Data Availability

Strain HC1292 is available at the CGC, the rest are available upon request. The authors affirm that all data necessary for confirming the conclusions of the article are present within the article, figures, tables and Supplementary file. The Supplementary file contains a summary table of screening pipeline outcomes as well as individual worksheets for the data underlying each figure and off-target sequencing results. Also included is the custom R script, Supplemental_Material_G3_-2025-405680.rmd, which was used to analyze sid-1 AG and UcrRNA_AW1 off-targets. Supplemental material available at G3 online.
